# Establishment and characterization of a rat model of scalp-cranial composite defect for multilayered tissue engineering

**DOI:** 10.21203/rs.3.rs-4643966/v1

**Published:** 2024-07-23

**Authors:** Yi Zhu, Ou Mei, Hui Zhang, Wulin You, Jiamin Zhong, Caralyn P. Collins, Guowei Shen, Changqi Luo, Xingye Wu, Jingjing Li, Yi Shu, Ya Wen, Hue H. Luu, Lewis L. Shi, Jiaming Fan, Tong-Chuan He, Guillermo A. Ameer, Cheng Sun, Liangyuan Wen, Russell R. Reid

**Affiliations:** The University of Chicago Medical Center; The University of Chicago Medical Center; The University of Chicago Medical Center; The University of Chicago Medical Center; The University of Chicago Medical Center; Northwestern University; The University of Chicago Medical Center; The University of Chicago Medical Center; The University of Chicago Medical Center; The University of Chicago Medical Center; The University of Chicago Medical Center; Capital Medical University; The University of Chicago Medical Center; The University of Chicago Medical Center; The University of Chicago Medical Center; The University of Chicago Medical Center; Northwestern University; Northwestern University; Chinese Academy of Medical Sciences & Peking Union Medical College; The University of Chicago Medical Center

**Keywords:** Composite defect, Scalp-cranial, Rat model, Autologous reconstruction

## Abstract

Composite cranial defects have individual functional and aesthetic ramifications, as well as societal burden, while posing significant challenges for reconstructive surgeons. Single-stage composite reconstruction of these deformities entail complex surgeries that bear many short- and long-term risks and complications. Current research on composite scalp-cranial defects is sparse and one-dimensional, often focusing solely on bone or skin. Thus, there is an unmet need for a simple, clinically relevant composite defect model in rodents, where there is a challenge in averting healing of the skin component via secondary intention. By utilizing a customizable (3D-printed) wound obturator, the scalp wound can be rendered non-healing for a long period (more than 6 weeks), with the cranial defect patent. The wound obturator shows minimal biotoxicity and will not cause severe endocranium-granulation adhesion. This composite defect model effectively slowed the scalp healing process and preserved the cranial defect, embodying the characteristics of a “chronic composite defect”. In parallel, an autologous reconstruction model was established as the positive control. This positive control exhibited reproducible healing of the skin within 3 weeks with variable degrees of osseointegration, consistent with clinical practice. Both models provide a stable platform for subsequent research not only for composite tissue engineering and scaffold design but also for mechanistic studies of composite tissue healing.

## INTRODUCTION

Composite cranial defects, which affect multiple tissue types, pose significant challenges for reconstructive surgeons ^1^. Etiologies of composite cranial defects are diverse, including penetrating trauma, burns, tumor resections, radiation exposure, or infections ^2^. Often, many of these cases cannot undergo one-stage repair due to factors such as concurrent infections, polytrauma, ongoing radiation therapy, or prior cranioplasty failures, thereby leading to chronic defects ^3^. Furthermore, single-stage reconstructive measures, such as composite or chimeric free-flaps, involve extensive undertakings fraught with potential risks and complications (references). Notably, the transition from acute to chronic repair significantly impacts healing dynamics, most prominently observed as a deceleration in healing speed ^4^ and a reduced success rate of autologous bone and soft tissue reconstruction^5,6^.

In recent decades, biomaterial-based cranial or scalp repair through tissue engineering has emerged as a focal point in research ^7^. Rodents, valued for their small size and ease of maintenance, serve as effective animal models extensively used in studies of cranial defects and skin regeneration ^8–11^. However, composite defect models, particularly those that mirror chronic clinical conditions, are rarely reported. The primary challenge is the rapid rate of skin healing in rodents, which can culminate within two weeks ^12^. This rapid healing hinders the investigation of chronic defect regeneration.

Existing models for chronic skin defects in rodents include: 1) Pressure ischemia-reperfusion, which involves multiple cycles of ischemia and reperfusion using a steel plate and magnet ^13^. This method is cumbersome and the metal components interfere with micro-CT scans for studies involving cranial defects; 2) Diabetic rodents, which require a prolonged period to develop the diabetic condition and do not accurately replicate clinical scenarios ^14^; and 3) Infection-based models, which are difficult to control and may adversely affect regenerative outcomes ^15^. None of these models are ideally suited for studying composite cranial defects in rodents.

In this study, we developed a clinically relevant, chronic composite cranial defect model in rats. Specifically, we successfully established a rat model of a scalp-cranial composite defect along with a unique complementary positive control, providing a stable platform for subsequent tissue repair and bone regeneration studies. The experimental model effectively slowed the scalp healing process and preserved the cranial defect, embodying the characteristics of a “chronic composite defect”. The positive control exhibited reliable and reproducible healing through autologous reconstruction, mirroring current surgical practice. Collectively, our reported model provides a stable platform for subsequent research not only for composite tissue engineering and scaffold design but also for mechanistic studies of composite tissue healing.

## MATERIALS AND METHODS

### Cell Culture and Chemicals

Human HEK-293 cells were obtained from the American Type Culture Collection (ATCC, Manassas, VA). 293pTP, RAPA and 293GP cells were derived from HEK-293 cells as previously described ^16–18^. The mouse mesenchymal cell line iMAD was previously described ^19,20^. All cells were cultured in DMEM supplemented with 10% fetal bovine serum (FBS, Gemini Bio-Products), 100 U/ml penicillin, and 100μg/ml streptomycin at 37°C in 5% CO_2_ as described ^21–26^. Unless indicated otherwise, chemicals were obtained from Thermo Fisher Scientific (Waltham, MA) or Millipore Sigma (St. Louis, MO).

### Construction and Amplification of the Recombinant Adenoviral Vector Ad-GFP-GLuc Expressing Both GFP and Gaussia Luciferase (GLuc)

Recombinant adenovirus Ad-GFP-GLuc was constructed by using either the AdEasy technology or the Gibson DNA Assembly-based OSCA system as previously described ^27–30^. Briefly, the coding region of Gaussia luciferase (GLuc) ^31–33^ was PCR amplified and subcloned into the adenoviral shuttle vector pAdTrack-CMV, followed by homologous recombination reactions with the adenoviral backbone vector pAdEasy1 in BJ5183 cells ^28,34^. The resultant recombinant adenoviral plasmid was used to generate adenovirus Ad-GFP-GLuc. Alternatively, the Ad-GFP-GLuc was constructed by using the Gibson DNA Assembly-based OSCA system as previously described ^30^. The recombinant adenovirus Ad-GFP-GLuc was packaged in 293pTP cells, and amplified to high titers in HEK-293, RAPA, 293pTP, or 293GP cells ^16–18^. The Ad-GFP-GLuc also co-expresses GFP marker gene. Polybrene (final concentration at 6μg/mL) was added to enhance adenoviral transduction efficiency in the iMAD cells as previously described ^35,36^.

### Surgical Procedures of Initial Operation

The protocols of this study were approved by the Institutional Animal Care and Use Committee (IACUC) of The University of Chicago (ACUP #71445). All experiments were performed in accordance with relevant guidelines and regulations. Bone and soft tissue defects in rat models (8-week-old Sprague Dawley rats, weight range 200–350g; Envigo, Indianapolis, IN) were carried out following the approved guidelines.

**Figure 1:**
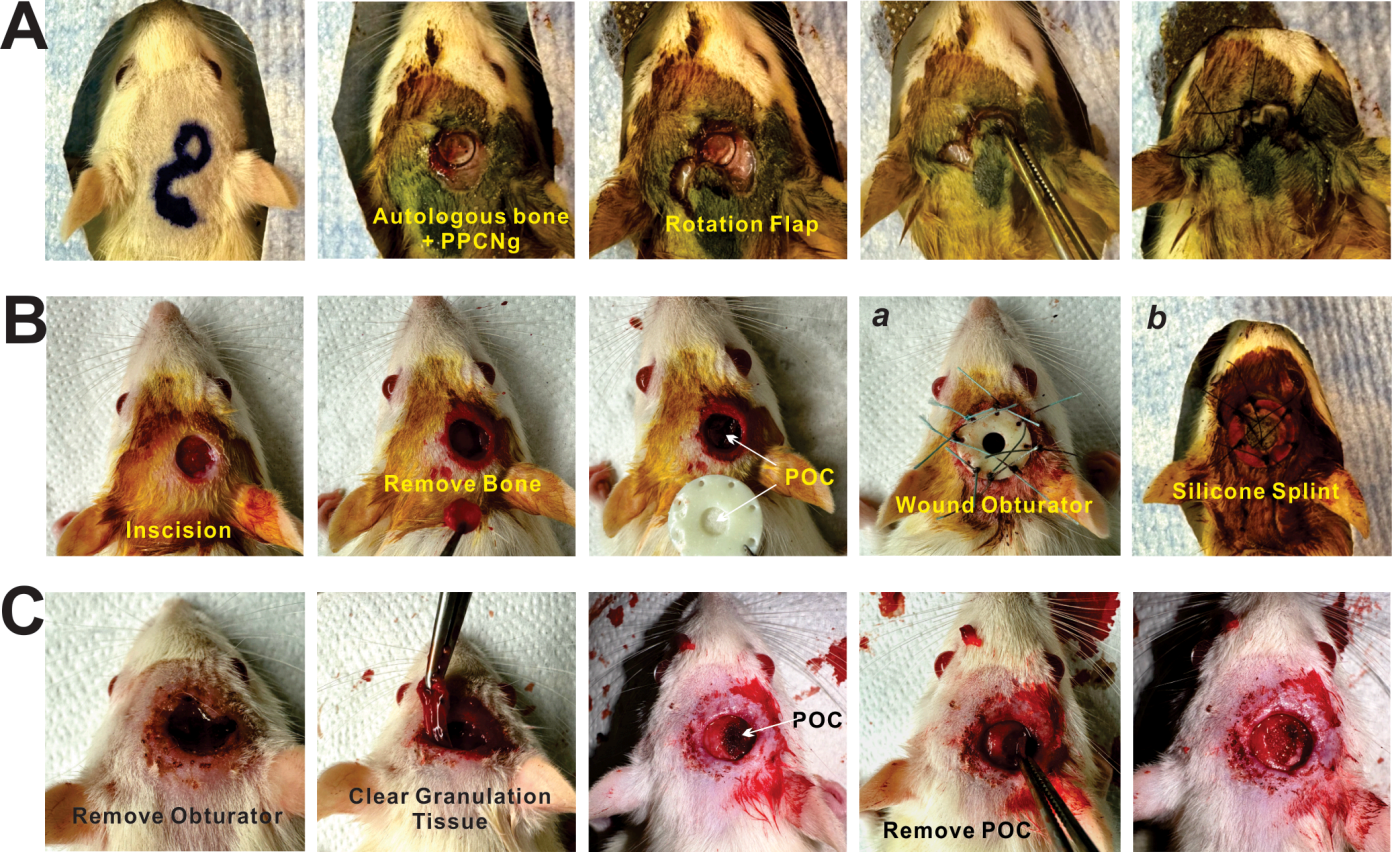
Surgical procedures for the creation of scalp-cranial bone defect. **(A)** In the positive control (autologous reconstruction) group, the excised bone was replaced as an autograft with 100μl glycol citrate-co-*N*-isopropylacrylamide gelatin (PPCNg), and the overlying wound was reconstructed using a rotation flap. **(B)** For negative controls, the incised scalp and cranial bone were removed, and the wound was merely wrapped in gauze to inhibit infection **(*a* & *b*).** However, in the experimental group, a citrate-based polymer poly (octamethylene citrate) (POC) disc was implanted into the cranial defect to prevent adhesion between the dura mater and granulation tissue **(*c*).** The incision was further supplemented using either a wound obturator **(*d*)** or a silicone splint **(*e*). (C)** Three weeks post-initial surgery, the experimental group (WO group) underwent a secondary operation for preparation of further regeneration research. Granulation and connective tissues were meticulously excised to refresh the margins of bone and scalp.

Images of the surgical procedures are shown in Fig. 1. Briefly, anesthesia was induced in rats through isoflurane inhalation, complemented by intraperitoneal injection of ketamine (50–75 mg/kg) and xylazine (7–10 mg/kg). After prone positioning, the rat was prepared by scalp depilation, sterilization with iodine tincture, and application of a sterile drape. Using a biopsy punch, a 6 mm circular incision was made above the parietal cranium, and the pericranium was removed with a neuro spatula. A cranial defect was then carefully drilled in the parietal bone with a 6 mm trephine under continuous normal saline irrigation to prevent tissue thermal injury (Dremel^®^ USA, Robert Bosch Tool Corp). The bone disc was removed, and hemostasis was meticulously achieved.

In the positive control (autologous reconstruction) group (n = 6, three for histological staining at 5 weeks, three for long-term micro-CT scan), the excised bone was replaced as an autograft with 100μl of a biocompatible, thermosensitive polymer, polyethylene glycol citrate-co-N-isopropylacrylamide pre-mixed with gelatin (PPCNg) ^37,38^, and the overlying wound was reconstructed using a rotation flap (Fig. 1A). Conversely, the negative control group (n = 3, one for histological staining at 5 weeks, two for long-term micro-CT scan) was left with the bone and scalp defects untreated; the wound was merely wrapped in gauze to inhibit infection. For the experimental group, the incision was supplemented using either a standard silicone splint (Grace Bio-Labs; n = 4) or a wound obturator (n = 6, two for histological staining at 3 weeks, 4 for secondary operation) to counteract the rapid initial self-healing of the scalp post-trauma ^39^ (Fig. 1B). To prevent adhesion between the dura mater and granulation tissue, a citrate-based polymer poly (octamethylene citrate) (POC) disc ^40^ was implanted into the cranial defect. In the experimental group, 100 μL of an anti-inflammatory mixture (1 μM vincristine, 0.5 mg/mL kanamycin) were injected through the central opening of the wound obturator (WO), designed for absorption and gradual release by the POC within the obturator. Vincristine’s application was aimed at inhibiting the granulation tissue generation process. All groups received standard postoperative pain relief and monitoring per IACUC guidelines.

### Surgical Procedures of Secondary Operation for Further Tissue Regeneration Study

Three weeks post-initial surgery, the experimental group (WO group) underwent a secondary operation (Fig. 1C). Following anesthesia and sterilization, all sutures were carefully removed along with the wound obturator. Mimicking clinical conditions, granulation and connective tissues were meticulously excised to refresh the margins of bone and scalp. This was achieved using hemostatic forceps, scissors, and scalpels in a layer-by-layer approach. Subsequently, the POC disc, which prevented adhesion between the dura mater and granulation tissue, was gently lifted and the margins of the cranial defect were cleared. By this secondary method, the surgically debrided defects are primed for skin-bone tissue engineering via regenerative scaffold placement.

### Wound Obturator (WO) Design and 3-D Printing

**Figure 2:**
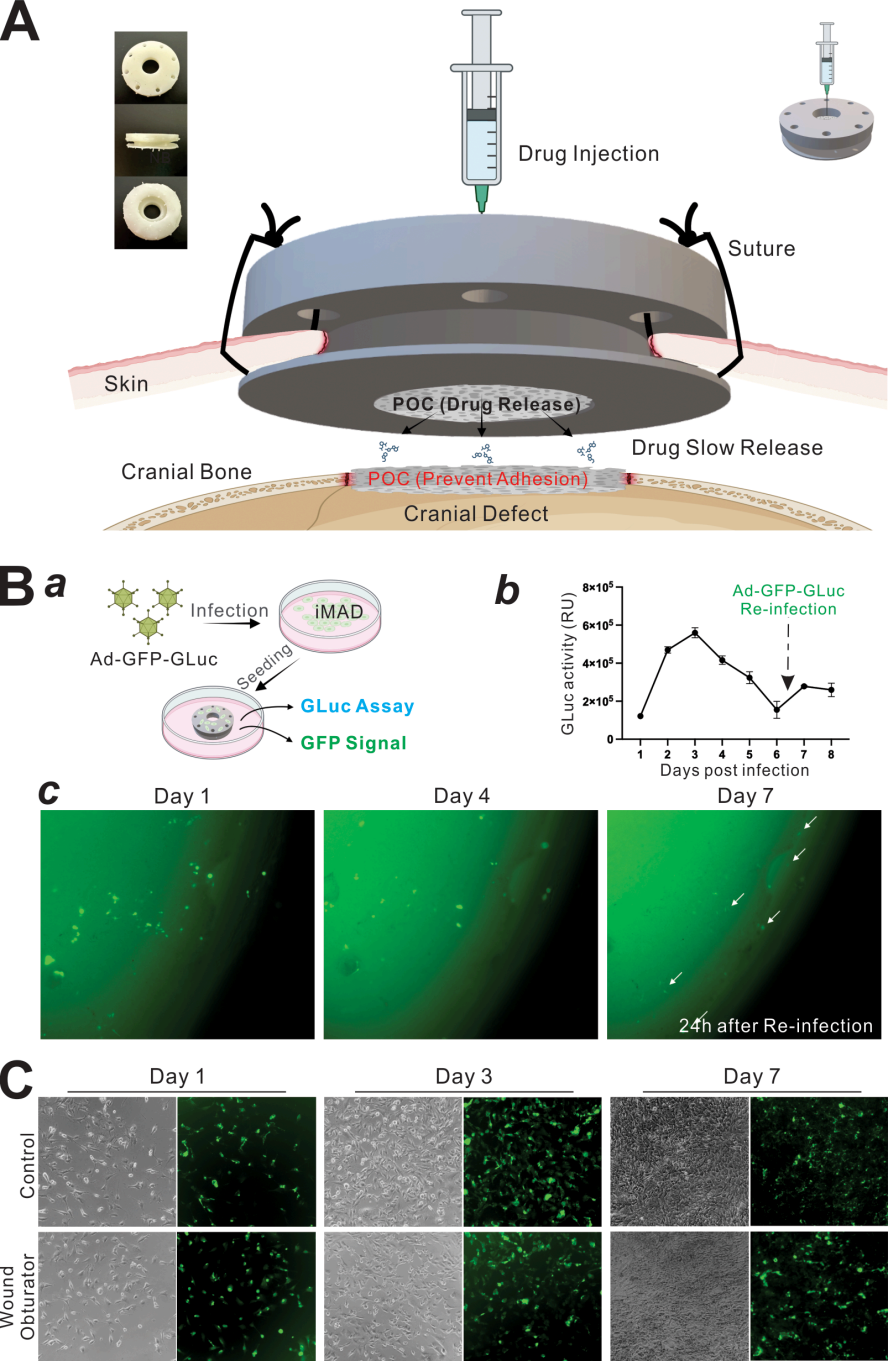
Wound Obturator (WO) design and cell compatibility assays. **(A)** Design of WO and its application in surgery. **(B)** Subconfluent iMAD cells were transduced with Ad-GFP-GLuc. Post-infection, cells were applied to WO submerged in culture medium. After 6 days, the cell-laden WO was reinfected with Ad-GFP-GLuc **(a)**. Both Gaussia luciferase activity **(b)** and GFP fluorescence **(c)** were monitored as indicators of cell viability. The white arrows indicate the re-infected cells. **(C)** Co-culture images of WO with iMADs. Viable cells were stained with Calcein-AM and imaged at the indicated time points. Representative results are shown.

The wound obturator utilized in the experimental group is depicted in Fig. 2A. This device, crafted from resin xFLEX475-white (NEXA 3D) through 3-D printing, comprises two rings that securely anchor the scalp, while an inner cylindrical wall offers mechanical resistance against centripetal scalp growth. A POC disc was placed within the central cavity. The upper ring features a central hole that facilitates both exudate drainage and serves as an access point for injecting therapeutics. Peripheral holes are designed for suturing. Both the POC and the obturator were sterilized via a two-hour immersion in PBS containing penicillin and streptomycin, followed by comprehensive UV post-curing.

### Cell-Wound Obturator Co-Culture Cell Viability Assay

Exponentially growing iMAD cells were seeded at a low density (5 × 10^4^ cells/well) in 12-well plates and co-cultured with either the wound obturator (WO) or no WO control. Viable cells were visualized by using Calcein-AM staining and imaged at indicated time points as previously described ^41,42^.

### Cell Biocompatibility Assay

Subconfluent iMAD cells were infected with Ad-GFP-GLuc for 16h. Cells were harvested, resuspended in DMEM, and seeded to the pre-treated obturators submerged in culture medium (approx. 1 × 10^4^ cells per seeding) (Fig. 2B–a). At 6 days post seeding, the cell-laden obturator was reinfected with Ad-GFP-GLuc. Both GFP fluorescence and the secreted Gaussia luciferase activity were monitored at the indicated timepoints as indicators of cell viability. Gaussia luciferase activity was quantified using the Secrete-Pair^™^ Gaussia Luciferase Assay Kit (GeneCopoeia, Rockville, MD) as previously described ^24,31,43^.

### Micro-CT Imaging Analysis

Live rats were subjected to micro-CT scans at weeks 0 (three days post-operation), 3, 5, and 12, except for the WO group, which was scanned at weeks 0, 3, and 5, using the X-CUBE Preclinical CT Imager (Molecubes NV, Belgium) at The University of Chicago Integrated Small Animal Imaging Research Resource (iSAIRR) facilities. Spiral high-resolution CT acquisitions were performed with an x-ray source of 50 kVp and 200 μA. Volumetric CT images were reconstructed in a 350 × 350 × 840 format with voxel dimensions of 200 μm^3^. A few volumetric CT images were also reconstructed in a 700 × 700 × 374 format with voxel dimensions of 100 μm^3^ to evaluate the performance of bone healing. Reconstructions were performed using 3-D Slicer software (Version 5.4.0) as previously described ^44–50^. Defect areas were quantified using Image J (Version 1.53k). Relative Cranial Defect Area Ratio (RCDAR) was calculated to quantify the healing rate. RCDAR= (Area at each timepoint/Area at week 0) *100%.

### Histological Evaluation

The retrieved cranial specimens were fixed in 10% PBS-buffered formalin for 2 days and decalcified in 5% nitric acid, followed by dehydration, paraffin embedding, and sectioning. Sections were deparaffinized and subjected to H & E staining (Volu-Sol) and Masson trichrome staining (Newcomer Supply) as previously described ^51–56^.

### Statistical Analysis

All experiments were performed at least three times or repeated in three batches of independent experiments. Data were analyzed using GraphPad Prism 7 and presented as the mean ± standard deviations (SD). Statistical significance was determined by one-way ANOVA and the student’s t-test for the comparisons between groups. A value of p < 0.05 was considered statistically significant. The work has been reported in line with the ARRIVE criteria ^57^.

## RESULTS

### Rapid skin healing prevents the creation of a rat model of clinically relevant scalp-cranial composite defect

For the establishment of a reliable murine composite scalp-cranial defect model, we had to devise both clinically relevant positive and negative controls. (Fig. 1). In the positive control (autologous reconstruction) group, the excised bone was replaced as an autograft with 100μl of the biocompatible, thermosensitive polymer PPCNg^37^, and the overlying wound was reconstructed using a rotation flap (Fig. 1A). Conversely, the negative control group was left with the bone and scalp defects untreated; the wound was merely covered in gauze to prevent desiccation (Fig. 1B–ab).

For the experimental group, a citrate-based polymer poly (octamethylene citrate) (POC) disc ^40^ was first implanted into the cranial defect to prevent adhesion between the dura mater and granulation tissue (Fig. 1B–c). Our preliminary experiments indicated that the 6-mm circular scalp wound in rats healed effectively in 7–10 days. Thus, to produce a non-healing composite wound, we further supplemented the defect with either a homemade wound obturator (WO) (Fig. 1B–d) or a silicone splint (Fig. 1B–e). While the silicone splint is commercially available and somewhat widely used, our results demonstrated that our homemade 3D printed wound obturator provided more rigid fixation and thus more effective prevention of scalp healing.

To mimic clinical conditions, the experimental group (WO group) underwent a second “priming” operation to prepare the composite defect for composite reconstruction via tissue engineering strategies (Fig. 1C). This priming procedure involved removal of the wound obturator, followed by meticulous sharp debridement of granulation tissue to refresh the margins of the bone and scalp. Subsequently, the POC disc, which prevented adhesion between the dura mater and granulation tissue, was gently lifted and the margins of the cranial defect were cleared (Fig. 1C).

### The 3-D printed Wound Obturator (WO) shows minimal biotoxicity and does not cause severe endocranium-granulation adhesion

As shown in Fig. 2A, the wound obturator device was crafted from resin xFLEX475-white (NEXA 3D) through 3-D printing, comprising two rings that securely anchor the scalp, while a inner cylindrical wall offers mechanical resistance against centripetal scalp growth. Within the central cavity, a POC disc was placed to serve the following functions: a) physically occupying space to stent the wound and prevent wound closure via contraction (“secondary intention”), and to minimize granulation tissue formation inside the central cavity; b) acting biochemically as a repository to absorb and gradually release medications, including antibiotics and agents that slow cell proliferation; and c) absorbing inflammatory exudates to protect against potential infections. The upper ring features a central hole that facilitates both exudate drainage and serves as an access point for injecting therapeutics. Peripheral holes are designed for suturing.

It is noteworthy that, to inhibit the granulation tissue generation process and prevent local infection, a mix of vincristine (1 μM) and kanamycin (0.5 mg/mL) was injected through the central opening for absorption and gradual release by the POC within the obturator (Fig. 2A). The POC was easily removable without any adhesion. Upon its removal, no invasion of fibrous tissue into the extradural space was observed, and the margins of the bone defect remained clear and clean, ideal for subsequent regenerative experimentation. In the 5-week histological section of the wound obturator group, the defect area remained protected by a distinct fibrous layer even after POC removal, preventing adhesion with granulation tissue after the secondary operation.

The biocompatibility assay demonstrated that Ad-GFP-Gluc infected iMAD cells could survive on the obturator for more than seven days (Fig. 2B–a). Upon reinfection with Ad-GFP-Gluc, Gaussia luciferase activity was partially restored (Fig. 2B–b) and emergent GFP signals were observed overnight (Fig. 2B–c). Furthermore, results from the cell-obturator co-culture assays revealed no significant differences in cell proliferation rates at days 3 and 7 (Fig. 2C), suggesting that the material used for 3-D printing of the wound obturator may exhibit little or no significant cytotoxicity.

### Autologous reconstruction achieves variable cranial bone healing in 3 weeks

**Figure 3:**
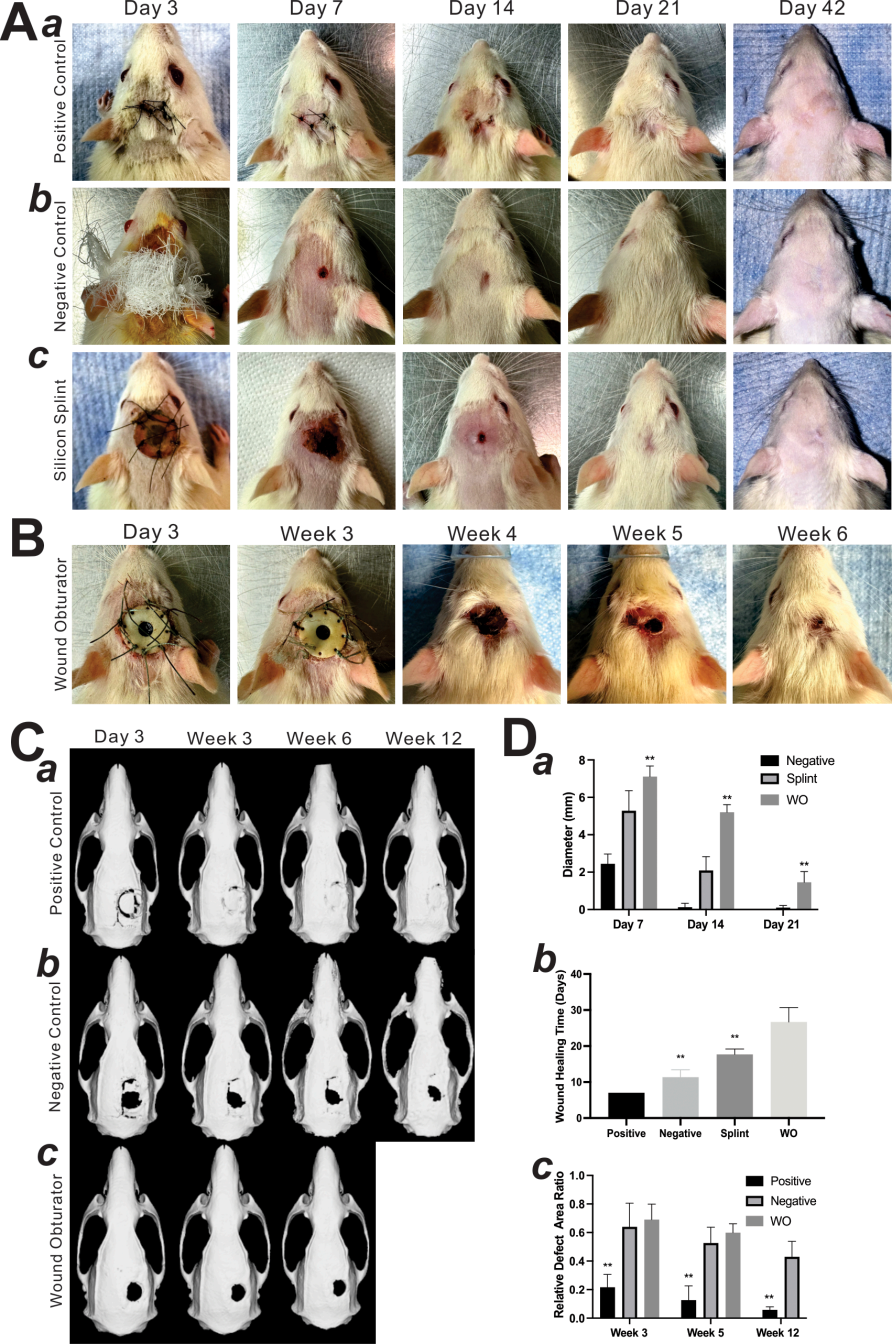
Scalp and cranial healing conditions at indicated time points. **(A)** Representative scalp healing images of the positive group (a), the negative group (b), the silicone splint group **(c)**, and the wound obturator (WO) group **(B)**. **(C)** Representative cranial healing 3D reconstruction images of the positive group (a), the negative group **(b)**, and the WO group **(c)**. **(D)** Data analysis graphs. Longer diameter of scab **(a)**, time points indicate post-initial operation for the negative group and splint group, while post-secondary operation for the WO group. Scalp complete healing time **(b).** Relative cranial defect area ratio (RCDAR) = (Area at each timepoint/Area at 0 week) *100% **(c)**. ”**” p<0.01, compared with that of the negative group.

As shown in Fig. 3A–a and Fig. 3C–a, the wound in the positive control group achieved complete healing within one week post operation. By the end of week 3, the positive control group exhibited retained bone graft take with varying amounts of osseointegration/fibrous union (RCDAR = 0.22 ± 0.09 at week 3), with a decelerated healing speed over the following weeks (RCDAR = 0.13 ± 0.10 at week 5, 0.06 ± 0.02 at week 12) (Fig. 3D).

**Figure 4:**
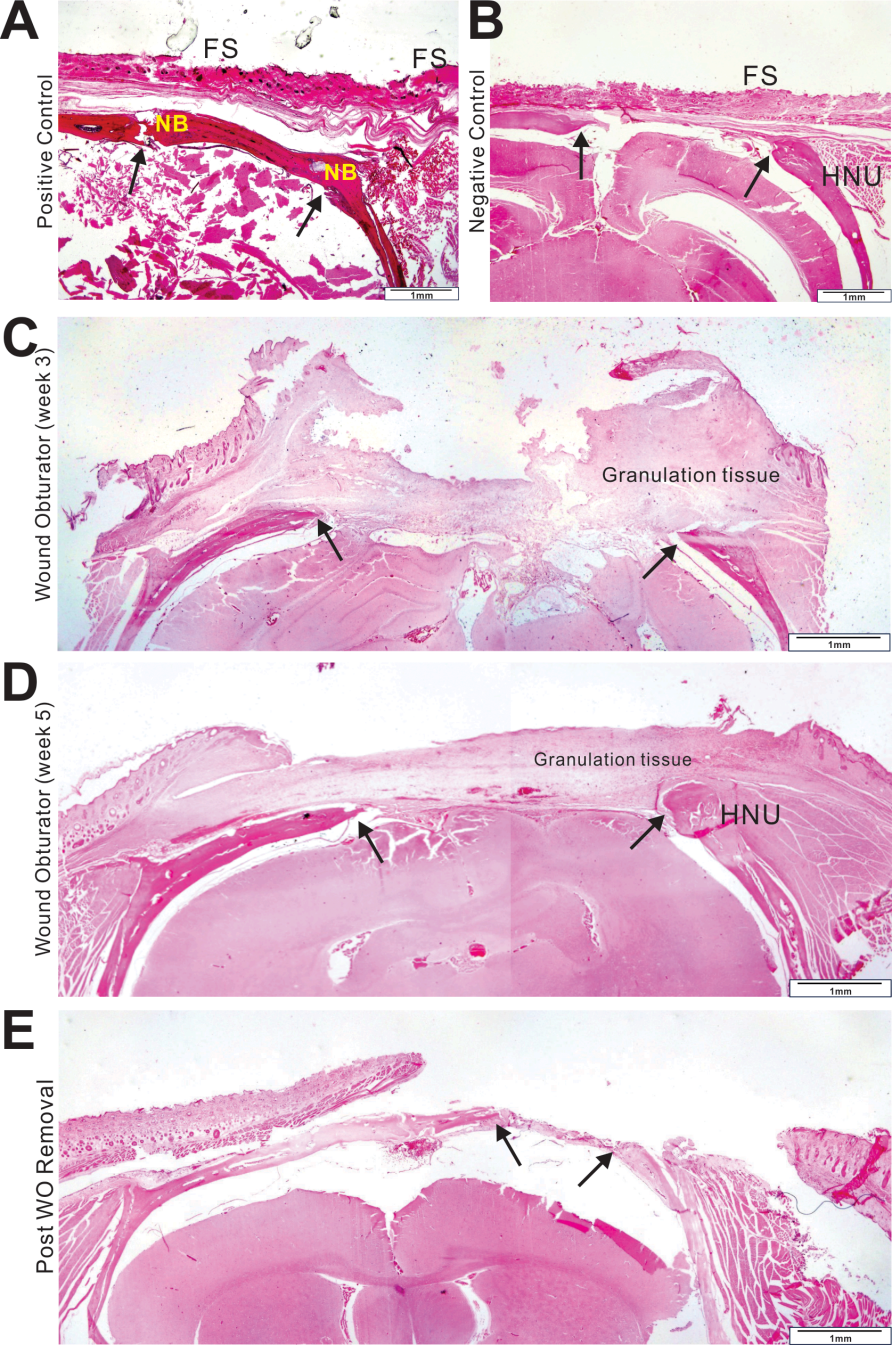
H & E staining of full-layer specimens. Full-layer specimens from positive control (**A**) and negative control (**B**) groups collected at 5 weeks, WO group (week 3) (**C**), WO group (week 5) (**D**) and Post WO removal (**E**) groups were fixed in 10% buffered formalin for 2 days, and decalcified in 5% nitric acid, followed by dehydration, paraffin embedding, and sectioning. Representative H & E staining results are shown. NB, new bone; FS, fibrous scar; HNU, hypertrophic non-union.

**Figure 5:**
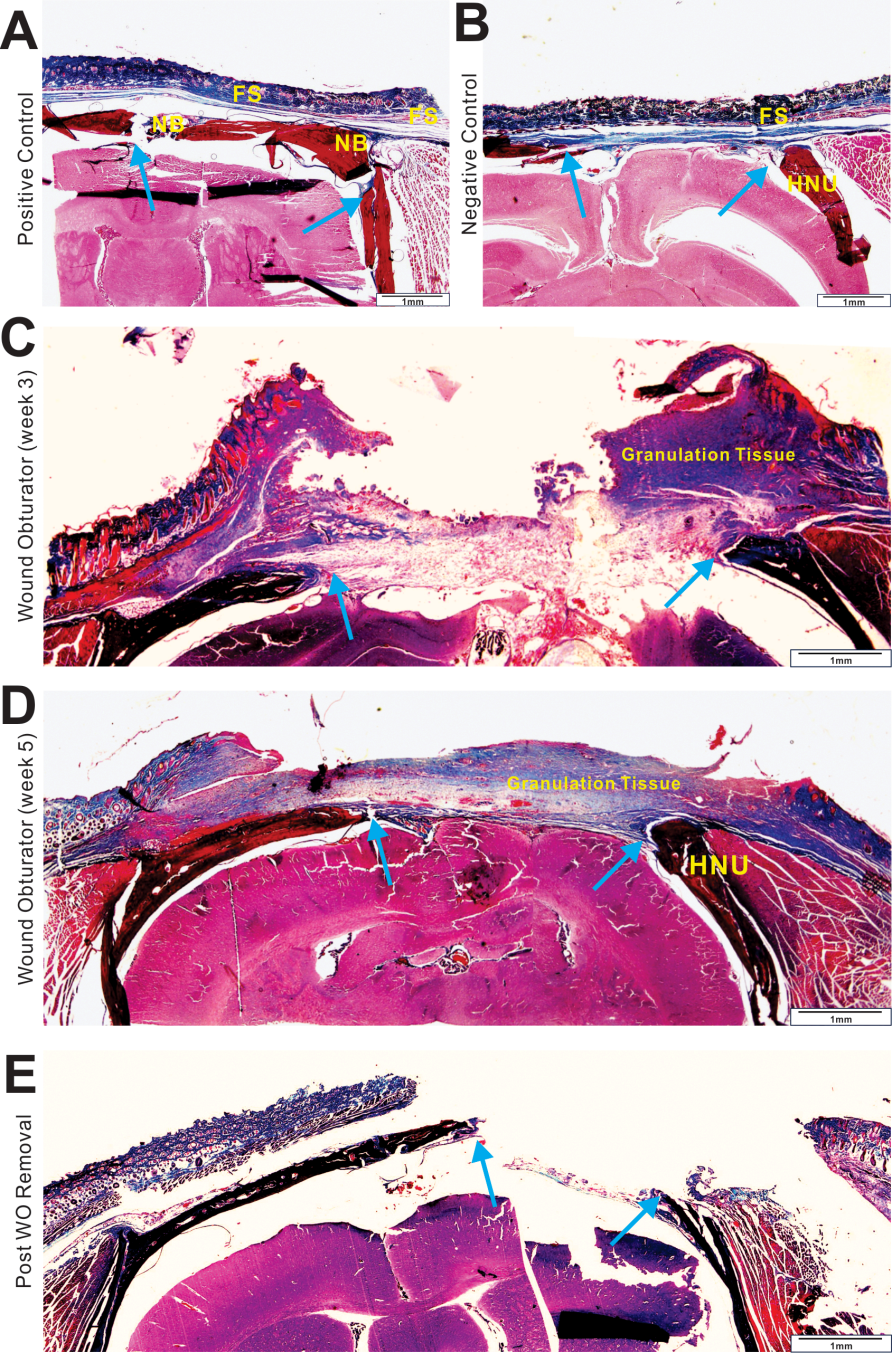
Trichrome staining of full-layer specimens. Full-layer specimens from positive control (**A**) and negative control (**B**) groups collected at 5 weeks, WO group (week 3) (**C**), WO group (week 5) (**D**) and Post WO removal (**E**) groups were processed as described in Figure 4, and subjected to trichrome staining analysis. Representative Trichrome staining results are shown. NB, new bone; FS, fibrous scar; HNU, hypertrophic non-union.

Histological evaluation indicated that in the positive group, bone healing was promoted by bone callus modeling, interspersed with fibrous connective tissue and nourishing blood vessels (Fig. 4A and Fig. 5A), characteristic of “secondary bone healing” ^57^. Regarding skin healing, the closure with a rotation flap resulted in complete coverage of the skin defect. No hypertrophic granulation tissue was observed.

### Critical-sized cranial defects exhibit limited bone self-healing capacity

In terms of animals in the negative control group, one in the silicone splint group was sacrificed because of uncontrollable infection, others survived until the endpoint. Micro-CT scans and subsequent 3D reconstructions revealed distinct cranial bone healing patterns among the groups (Fig. 3A–b and Fig. 3C–b). In contrast to the positive control group, the negative control group demonstrated markedly slower healing, showing an RCDAR of 0.64 ± 0.17, 0.53 ± 0.11, and 0.43 ± 0.11 at week 3, week 5, and week 12, respectively. Similarly, the WO group paralleled the negative control, with an RCDAR of 0.68 ± 0.02 at week 3 and 0.58 ± 0.06 at week 5 (Fig. 3D). These findings underscore the limited self-healing capacity of 6 mm cranial defects in rats without treatment over 12 weeks.

Histological analysis shows that the bony margins of the osseous defects in the negative group expanded into a round shape reminiscent of “hypertrophic nonunion” in clinical scenarios (Fig. 4B and Fig. 5B), thereby delaying or inhibiting the self-healing process ^58^. In the experimental group (WO group), the hypertrophic bone margin was not fully evident at 3 weeks post-trauma but became pronounced and consistent with that of the negative group by 12 weeks, suggesting that the first 3 to 4 weeks post-trauma may be critical for rapid bone healing.

### WO effectively delays skin self-healing capacity up to 5 weeks post surgery

Wound healing conditions are shown in Fig. 3B and Fig. 3C. In the negative control (non-splinted) group, the wound achieved complete healing by 11.33 ± 2.08 days (Fig. 3D–b). In the experimental group, a silicone splint was employed to keep the wound open but dislodged after around 6 days, resulting in healing within 17.67 ± 1.53 days post-operation (Fig. 3A–c and Fig. 3D–b). Compared to the negative group, wound healing of the silicone group was only delayed by about 6 days (Fig. 3A–c and Fig. 3D–a). In contrast, the wound obturator can be maintained for over 5 weeks. Additionally, after the removal of the obturator, wound healing duration was extended to 26.67 ± 4.04 days (Fig. 3D–b), which is approximately 15 days longer than the non-splinted control group, fitting the criteria of a non-healing, chronic wound.

Histological survey revealed that in the negative control group, the fibrous scar tissue significantly contracted into a smaller structure (Fig. 4B and Fig. 5B). For the wound obturator group at 3 weeks, the obturator had been freshly removed, thus presenting a chaotic histological phenotype, characterized by irregular fibrous scars and granulation tissue (Fig. 4C and Fig. 5C). By 5 weeks, however, the tissue appeared more organized and anatomically defined. The scalp defect was covered by granulation and fibrous tissue, with skin progressively enveloping this tissue, culminating in healing marked by a smaller wound (Fig. 4D and Fig. 5D).

## DISCUSSION

In the clinical arena, composite scalp-cranial defects arising from penetrating trauma, burns, tumor resections, radiation, or infections significantly compromise patient function and aesthetics, while producing a huge societal burden ^59^. The heterogeneity of these conditions demands considerable resources and versatility from reconstructive surgical teams to manage such complex injuries ^5^. Currently, the gold standard for reconstruction involves the use of autologous materials such as nonvascularized bone grafts, free soft tissue transfers, or combinations of vascularized bone and soft tissue (free composite or chimeric flaps) ^60^.

However, many of these cases cannot undergo one-stage repair due to factors such as concurrent infections, polytrauma, ongoing radiation therapy, or prior cranioplasty failures, thereby leading to “chronic defects” ^3,60^. Furthermore, the concept of replacing “like-with-like” in these defects may demand a large donor-site burden. Biomaterial-based reconstruction with cellular-based tissue engineering strategies represents a viable alternative in such circumstances, but it requires extensive validation before clinical adoption ^61^. Despite the clinical impact, research on composite scalp-cranial defects is sparse, often focusing solely on bone or skin. Thus, there is a clear need for a simple, clinically relevant composite defect model to serve as a testbed for regenerative approaches.

We established an autologous reconstruction model as the positive control, closely aligned with clinical practice. After replanting the cranial bone and utilizing a rotational flap for wound closure, rapid multilayer healing was observed within 3 weeks postoperatively. PPCNg ^37^, a thermoresponsive biomaterial that transitions reversibly from liquid to solid at 37°C, was applied to provide semi-rigid fixation of the bone graft and potentially accelerate the healing process. The PPCNg has been shown to promote wound closure without inducing a significant inflammatory response ^62^. However, it is important to note that although composite wound healing is achieved under these conditions, variations in autologous bone graft “take” and osseointegration in our model, which mimics clinical conditions, demand regenerative strategies to improve composite tissue healing. Additionally, in the clinical realm, often autologous options are not available or feasible due to patient factors. Nevertheless, the creation of a rodent positive control that mimics the “gold standard” is a novel feature of our project and establishes a benchmark to which to compare all other therapeutic approaches.

The composite scalp-cranial defect model is a key focus of our study. The primary challenge is maintaining the scalp wound in an unhealed state for a longer duration. Previously, several approaches have been developed to establish chronic skin conditions in rodents. Chen et al. ^62^ created ischemic wounds within a bi-pedicled dorsal flap using six uniformly placed incisions, demonstrating that wounds in non-necrotic ischemic zones heal more slowly than those on normally perfused skin; however, these effects were transient, delaying only 4 days more than control group, and the technique is unsuitable for used on head. Peirce et al. ^13^ surgically implanted a metal plate beneath the skin and applied periodic compressions using an external magnet. This method allows control over the size and severity of the injury by varying the number and duration of compressions, replicating features of human chronic wounds such as reduced blood flow, hypoxia, and immune cell influx. However, this technique is unsuitable for cranial defect studies due to interference with micro-CT scans from the metal. While diabetes models are beneficial for studying diabetes-related wounds, they may not be appropriate for trauma studies ^14,63,64^. Infection models, although relevant, pose challenges in controlling outcomes and may negatively impact further regeneration studies ^15^.

Our approach employs mechanical resistance to counteract wound centripetal contraction. Over time, chronic wounds exhibit significantly elevated levels of proinflammatory cytokines and matrix metalloproteinases, whereas activities of matrix metalloproteinase inhibitors and growth factors are reduced, thereby decelerating the healing process ^65–67^. Compared to previously described methods, our method is simpler to implement, more closely aligns with trauma-related composite defects, and effectively extends the duration of a non-healing wound.

In addition to mirroring clinical practice, ease of operation is crucial for animal models; rodents provide this advantage, both in terms of husbandry and surgical procedures. Rodents also provide the advantage of being genetically modifiable and thus the opportunity to evaluate knockout or knock-in effects of essential signaling pathways on composite tissue healing and bone regeneration ^68–77^.

To inhibit the proliferation of adherent granulation tissue during the splinting process, we employed POC as a barrier and a bio-release medium, combined with Vincristine (cell proliferation decelerator) and Kanamycin (antibiotics). POC has been demonstrated to have minimal cytotoxicity and immune response but increased cell compatibility. In our study, POC did not elicit any related adverse reactions. Instead, it significantly reduced dural adhesions, thereby simplifying the surgical environment for subsequent research.

The longest remaining duration of WO tested was 5 weeks, which showed no difference in wound healing speed between the obturator removed at 5 weeks and those removed at 3 weeks. Thus, we surmise that 3 weeks is sufficient for the obturator to bypass the peak healing period of the skin. This model, capable of maintaining a non-healing state for extended periods, is suitable not only for the development of regenerative strategies but also for investigating inflammatory pathways, and repair mechanisms of chronic composite defects.

## CONCLUSION

We have successfully established a rat model of a scalp-cranial composite defect along with a unique complementary positive control, providing a stable platform for subsequent research. The experimental model effectively slowed the scalp healing process and preserved the cranial defect, embodying the characteristics of a “chronic composite defect”. The positive control exhibited reliable and reproducible healing through autologous reconstruction, mirroring current surgical practice.

## Data Availability

All data are available in the main text or the supplementary materials.

## References

[1] Lee J. C., Kleiber G. M., Pelletier A. T., Reid R. R. & Gottlieb L. J. Autologous immediate cranioplasty with vascularized bone in high-risk composite cranial defects. Plast Reconstr Surg 132, 967–975, doi:10.1097/PRS.0b013e31829f4b59 (2013). 24076686

[2] Soto E., Restrepo R. D., Grant J. H. 3rd & Myers R. P. Outcomes of Cranioplasty Strategies for High-Risk Complex Cranial Defects: A 10-Year Experience. Ann Plast Surg 88, S449–S454, doi:10.1097/SAP.0000000000003019 (2022). 34670972 PMC8986876

[3] Fong A. J. Reconstructive approach to hostile cranioplasty: A review of the University of Chicago experience. J Plast Reconstr Aesthet Surg 68, 1036–1043, doi:10.1016/j.bjps.2015.04.014 (2015). 25971417

[4] Han G. & Ceilley R. Chronic Wound Healing: A Review of Current Management and Treatments. Adv Ther 34, 599–610, doi:10.1007/s12325-017-0478-y (2017). 28108895 PMC5350204

[5] Shonka D. C. Jr., Potash A. E., Jameson M. J. & Funk G. F. Successful reconstruction of scalp and skull defects: lessons learned from a large series. Laryngoscope 121, 2305–2312, doi:10.1002/lary.22191 (2011). 22020883

[6] Shimizu F. Algorithm for reconstruction of composite cranial defects using the fascial component of free anterolateral thigh flaps. J Craniofac Surg 24, 1631–1635, doi:10.1097/SCS.0b013e3182999a33 (2013). 24036741

[7] Szpalski C., Barr J., Wetterau M., Saadeh P. B. & Warren S. M. Cranial bone defects: current and future strategies. Neurosurg Focus 29, E8, doi:10.3171/2010.9.FOCUS10201 (2010). 21121722

[8] Wang H. A 3D biomimetic optoelectronic scaffold repairs cranial defects. Sci Adv 9, eabq7750, doi:10.1126/sciadv.abq7750 (2023). 36791200 PMC9931229

[9] Kwon D. Y. Bone regeneration by means of a three-dimensional printed scaffold in a rat cranial defect. J Tissue Eng Regen Med 12, 516–528, doi:10.1002/term.2532 (2018). 28763610

[10] Yu C. A novel microcurrent dressing for wound healing in a rat skin defect model. Mil Med Res 6, 22, doi:10.1186/s40779-019-0213-x (2019). 31331385 PMC6647105

[11] Rashtbar M. Critical-sized full-thickness skin defect regeneration using ovine small intestinal submucosa with or without mesenchymal stem cells in rat model. Journal of Biomedical Materials Research Part B-Applied Biomaterials 106, 2177–2190, doi:10.1002/jbm.b.34019 (2018). 29052357

[12] Masson-Meyers D. S. Experimental models and methods for cutaneous wound healing assessment. Int J Exp Pathol 101, 21–37, doi:10.1111/iep.12346 (2020). 32227524 PMC7306904

[13] Peirce S. M., Skalak T. C. & Rodeheaver G. T. Ischemia-reperfusion injury in chronic pressure ulcer formation: a skin model in the rat. Wound Repair Regen 8, 68–76, doi:10.1046/j.1524-475x.2000.00068.x (2000). 10760216

[14] Fang R. C. Limitations of the db/db mouse in translational wound healing research: Is the NONcNZO10 polygenic mouse model superior? Wound repair and regeneration 18, 605–613 (2010). 20955341 10.1111/j.1524-475X.2010.00634.x

[15] Tachi M., Hirabayashi S., Yonehara Y., Suzuki Y. & Bowler P. Development of an experimental model of infected skin ulcer. Int Wound J 1, 49–55, doi:10.1111/j.1742-481x.2004.00006.x (2004). 16722897 PMC7951774

[16] Wu N. Overexpression of Ad5 precursor terminal protein accelerates recombinant adenovirus packaging and amplification in HEK-293 packaging cells. Gene Ther 21, 629–637, doi:10.1038/gt.2014.40 (2014). 24784448

[17] Wei Q. Engineering the Rapid Adenovirus Production and Amplification (RAPA) Cell Line to Expedite the Generation of Recombinant Adenoviruses. Cell Physiol Biochem 41, 2383–2398, doi:10.1159/000475909 (2017). 28463838

[18] Zhao G. GAPDH suppresses adenovirus-induced oxidative stress and enables a superfast production of recombinant adenovirus. Genes & Diseases, 101344, doi:10.1016/j.gendis.2024.101344 (2024). PMC11345542 39188753

[19] Lu S. Bone morphogenetic protein 9 (BMP9) induces effective bone formation from reversibly immortalized multipotent adipose-derived (iMAD) mesenchymal stem cells. Am J Transl Res 8, 3710–3730 (2016). 27725853 PMC5040671

[20] Gou Y. Adipose-derived mesenchymal stem cells (MSCs) are a superior cell source for bone tissue engineering. Bioact Mater 34, 51–63, doi:10.1016/j.bioactmat.2023.12.003 (2024). 38186960 PMC10770370

[21] Dong X. A simplified noncryogenic strategy to transport mesenchymal stem cells: Potential applications in cell therapy and regenerative medicine. Genes Dis 11, 101073, doi:10.1016/j.gendis.2023.07.002 (2024). 38274386 PMC10808911

[22] Guo M. Syrosingopine, an anti-hypertensive drug and lactate transporter (MCT1/4) inhibitor, activates hepatic stellate cells and exacerbates liver fibrosis in a mouse model. Genes Dis 11, 101169, doi:10.1016/j.gendis.2023.101169 (2024). 38434753 PMC10909599

[23] Zhang L. Transcriptomic landscape regulated by the 14 types of bone morphogenetic proteins (BMPs) in lineage commitment and differentiation of mesenchymal stem cells (MSCs). Genes Dis 6, 258–275, doi:10.1016/j.gendis.2019.03.008 (2019). 32042865 PMC6997588

[24] Zhang L. Modeling lung diseases using reversibly immortalized mouse pulmonary alveolar type 2 cells (imPAC2). Cell Biosci 12, 159, doi:10.1186/s13578-022-00894-4 (2022). 36138472 PMC9502644

[25] Zhong J. BMP4 upregulates glycogen synthesis through the SMAD/SLC2A1 (GLUT1) signaling axis in hepatocellular carcinoma (HCC) cells. Cancer Metab 11, 9, doi:10.1186/s40170-023-00310-6 (2023). 37443106 PMC10339511

[26] Huang L. Niclosamide (NA) overcomes cisplatin resistance in human ovarian cancer. Genes Dis 10, 1687–1701, doi:10.1016/j.gendis.2022.12.005 (2023). 37397523 PMC10311098

[27] He T. C. A simplified system for generating recombinant adenoviruses. Proc Natl Acad Sci U S A 95, 2509–2514, doi:10.1073/pnas.95.5.2509 (1998). 9482916 PMC19394

[28] Luo J. A protocol for rapid generation of recombinant adenoviruses using the AdEasy system. Nat Protoc 2, 1236–1247, doi:10.1038/nprot.2007.135 (2007). 17546019

[29] Lee C. S. Adenovirus-Mediated Gene Delivery: Potential Applications for Gene and Cell-Based Therapies in the New Era of Personalized Medicine. Genes Dis 4, 43–63, doi:10.1016/j.gendis.2017.04.001 (2017). 28944281 PMC5609467

[30] Ni N. A one-step construction of adenovirus (OSCA) system using the Gibson DNA Assembly technology. Mol Ther Oncolytics 23, 602–611, doi:10.1016/j.omto.2021.11.011 (2021). 34977337 PMC8666640

[31] Zhao C. A pH-Triggered, Self-Assembled, and Bioprintable Hybrid Hydrogel Scaffold for Mesenchymal Stem Cell Based Bone Tissue Engineering. ACS Appl Mater Interfaces 11, 8749–8762, doi:10.1021/acsami.8b19094 (2019). 30734555 PMC6407040

[32] Zhao C. Thermoresponsive Citrate-Based Graphene Oxide Scaffold Enhances Bone Regeneration from BMP9-Stimulated Adipose-Derived Mesenchymal Stem Cells. ACS Biomater Sci Eng 4, 2943–2955, doi:10.1021/acsbiomaterials.8b00179 (2018). 30906855 PMC6425978

[33] Zou Y. Gelatin-Derived Graphene-Silicate Hybrid Materials Are Biocompatible and Synergistically Promote BMP9-Induced Osteogenic Differentiation of Mesenchymal Stem Cells. ACS Appl Mater Interfaces 9, 15922–15932, doi:10.1021/acsami.7b00272 (2017). 28406027

[34] He T. C. Adenoviral vectors. Curr Protoc Hum Genet Chapter 12, Unit 12 14, doi:10.1002/0471142905.hg1204s40 (2004). 18428355

[35] Zhao C. Adenovirus-mediated gene transfer in mesenchymal stem cells can be significantly enhanced by the cationic polymer polybrene. PLoS One 9, e92908, doi:10.1371/journal.pone.0092908 (2014). 24658746 PMC3962475

[36] Yu Y. SV40 large T antigen-induced immortalization reprograms mouse cardiomyocyte progenitors with mesenchymal stem cell characteristics and osteogenic potential. Genes Dis 10, 1161–1164, doi:10.1016/j.gendis.2022.10.008 (2023). 37397535 PMC10311047

[37] Yang J., van Lith R., Baler K., Hoshi R. A. & Ameer G. A. A thermoresponsive biodegradable polymer with intrinsic antioxidant properties. Biomacromolecules 15, 3942–3952, doi:10.1021/bm5010004 (2014). 25295411

[38] Ye J. A thermoresponsive polydiolcitrate-gelatin scaffold and delivery system mediates effective bone formation from BMP9-transduced mesenchymal stem cells. Biomed Mater 11, 025021, doi:10.1088/1748-6041/11/2/025021 (2016). 27097687

[39] Dorsett-Martin W. A. Rat models of skin wound healing: a review. Wound Repair Regen 12, 591–599, doi:10.1111/j.1067-1927.2004.12601.x (2004). 15555049

[40] Ma C. In vitro cytocompatibility evaluation of poly(octamethylene citrate) monomers toward their use in orthopedic regenerative engineering. Bioact Mater 3, 19–27, doi:10.1016/j.bioactmat.2018.01.002 (2018). 29744439 PMC5935768

[41] Chen L. Insulin-like growth factor 2 (IGF-2) potentiates BMP-9-induced osteogenic differentiation and bone formation. J Bone Miner Res 25, 2447–2459, doi:10.1002/jbmr.133 (2010). 20499340 PMC3179288

[42] Huang E. Growth hormone synergizes with BMP9 in osteogenic differentiation by activating the JAK/STAT/IGF1 pathway in murine multilineage cells. J Bone Miner Res 27, 1566–1575, doi:10.1002/jbmr.1622 (2012). 22467218

[43] Zhong J. Reversibly immortalized keratinocytes (iKera) facilitate re-epithelization and skin wound healing: Potential applications in cell-based skin tissue engineering. Bioact Mater 9, 523–540, doi:10.1016/j.bioactmat.2021.07.022 (2022). 34820586 PMC8581279

[44] Shenaq D. S. Characterization of Reversibly Immortalized Calvarial Mesenchymal Progenitor Cells. J Craniofac Surg 26, 1207–1213, doi:10.1097/SCS.0000000000001717 (2015). 26080159 PMC4470299

[45] Mao Y. Argonaute (AGO) proteins play an essential role in mediating BMP9-induced osteogenic signaling in mesenchymal stem cells (MSCs). Genes Dis 8, 918–930, doi:10.1016/j.gendis.2021.04.004 (2021). 34522718 PMC8427325

[46] Luo W. BMP9-initiated osteogenic/odontogenic differentiation of mouse tooth germ mesenchymal cells (TGMCS) requires Wnt/beta-catenin signalling activity. J Cell Mol Med 25, 2666–2678, doi:10.1111/jcmm.16293 (2021). 33605035 PMC7933933

[47] Zhang B. Leptin Potentiates BMP9-Induced Osteogenic Differentiation of Mesenchymal Stem Cells Through the Activation of JAK/STAT Signaling. Stem Cells Dev 29, 498–510, doi:10.1089/scd.2019.0292 (2020). 32041483 PMC7153647

[48] Zhang Z. lncRNA Rmst acts as an important mediator of BMP9-induced osteogenic differentiation of mesenchymal stem cells (MSCs) by antagonizing Notch-targeting microRNAs. Aging (Albany NY) 11, 12476–12496, doi:10.18632/aging.102583 (2019). 31825894 PMC6949095

[49] Song D. BMP9 induces osteogenesis and adipogenesis in the immortalized human cranial suture progenitors from the patent sutures of craniosynostosis patients. J Cell Mol Med 21, 2782–2795, doi:10.1111/jcmm.13193 (2017). 28470873 PMC5661262

[50] He F. FAMSi: A Synthetic Biology Approach to the Fast Assembly of Multiplex siRNAs for Silencing Gene Expression in Mammalian Cells. Mol Ther Nucleic Acids 22, 885–899, doi:10.1016/j.omtn.2020.10.007 (2020). 33230483 PMC7658575

[51] Shu Y. Reversibly immortalized human umbilical cord-derived mesenchymal stem cells (UC-MSCs) are responsive to BMP9-induced osteogenic and adipogenic differentiation. J Cell Biochem 119, 8872–8886, doi:10.1002/jcb.27140 (2018). 30076626 PMC6195452

[52] Fan J. A simplified system for the effective expression and delivery of functional mature microRNAs in mammalian cells. Cancer Gene Ther 27, 424–437, doi:10.1038/s41417-019-0113-y (2020). 31222181 PMC6923634

[53] Fan J. Noncanonical Wnt signaling plays an important role in modulating canonical Wnt-regulated stemness, proliferation and terminal differentiation of hepatic progenitors. Oncotarget 8, 27105–27119, doi:10.18632/oncotarget.15637 (2017). 28404920 PMC5432321

[54] Zhang H. Canonical Wnt signaling acts synergistically on BMP9-induced osteo/odontoblastic differentiation of stem cells of dental apical papilla (SCAPs). Biomaterials 39, 145–154, doi:10.1016/j.biomaterials.2014.11.007 (2015). 25468367 PMC4258144

[55] Hu X. CRISPR/Cas9-mediated reversibly immortalized mouse bone marrow stromal stem cells (BMSCs) retain multipotent features of mesenchymal stem cells (MSCs). Oncotarget 8, 111847–111865, doi:10.18632/oncotarget.22915 (2017). 29340096 PMC5762364

[56] Cui J. BMP9-induced osteoblastic differentiation requires functional Notch signaling in mesenchymal stem cells. Lab Invest 99, 58–71, doi:10.1038/s41374-018-0087-7 (2019). 30353129 PMC6300564

[57] Cottrell J. A., Turner J. C., Arinzeh T. L. & O’Connor J. P. The Biology of Bone and Ligament Healing. Foot Ankle Clin 21, 739–761, doi:10.1016/j.fcl.2016.07.017 (2016). 27871408

[58] Rodriguez-Merchan E. C. & Forriol F. Nonunion: general principles and experimental data. Clin Orthop Relat Res 419, 4–12 (2004). 15021125

[59] Manson P. N., Crawley W. A. & Hoopes J. E. Frontal cranioplasty: risk factors and choice of cranial vault reconstructive material. Plast Reconstr Surg 77, 888–904 (1986). 3520618

[60] Afifi A. Lessons learned reconstructing complex scalp defects using free flaps and a cranioplasty in one stage. J Craniofac Surg 21, 1205–1209, doi:10.1097/SCS.0b013e3181e17c1e (2010). 20613618

[61] Tevlin R. Biomaterials for craniofacial bone engineering. J Dent Res 93, 1187–1195, doi:10.1177/0022034514547271 (2014). 25139365 PMC4237632

[62] Chen C. Molecular and mechanistic validation of delayed healing rat wounds as a model for human chronic wounds. Wound Repair Regen 7, 486–494, doi:10.1046/j.1524-475x.1999.00486.x (1999). 10633008

[63] Bannon P. Diabetes induces stable intrinsic changes to myeloid cells that contribute to chronic inflammation during wound healing in mice. Disease Models & Mechanisms 6, 1434–1447, doi:10.1242/dmm.012237 (2013). 24057002 PMC3820266

[64] Michaels J. t. db/db mice exhibit severe wound-healing impairments compared with other murine diabetic strains in a silicone-splinted excisional wound model. Wound Repair Regen 15, 665–670, doi:10.1111/j.1524-475X.2007.00273.x (2007). 17971012

[65] Goldman R. Growth factors and chronic wound healing: past, present, and future. Adv Skin Wound Care 17, 24–35, doi:10.1097/00129334-200401000-00012 (2004). 14752324

[66] Robson M. C. The role of growth factors in the healing of chronic wounds. Wound Repair Regen 5, 12–17, doi:10.1046/j.1524-475X.1997.50106.x (1997). 16984452

[67] Barrientos S., Stojadinovic O., Golinko M. S., Brem H. & Tomic-Canic M. Growth factors and cytokines in wound healing. Wound Repair Regen 16, 585–601, doi:10.1111/j.1524-475X.2008.00410.x (2008). 19128254

[68] Coalson E. Stem cell therapy for chronic skin wounds in the era of personalized medicine: From bench to bedside. Genes Dis 6, 342–358, doi:10.1016/j.gendis.2019.09.008 (2019). 31832514 PMC6888708

[69] Teven C. M., Fisher S., Ameer G. A., He T. C. & Reid R. R. Biomimetic approaches to complex craniofacial defects. Ann Maxillofac Surg 5, 4–13, doi:10.4103/2231-0746.161044 (2015). 26389027 PMC4555947

[70] Teven C. M. Differentiation of osteoprogenitor cells is induced by high-frequency pulsed electromagnetic fields. J Craniofac Surg 23, 586–593, doi:10.1097/SCS.0b013e31824cd6de (2012). 22446422

[71] Zhang F. Wnt and BMP Signaling Crosstalk in Regulating Dental Stem Cells: Implications in Dental Tissue Engineering. Genes Dis 3, 263–276, doi:10.1016/j.gendis.2016.09.004 (2016). 28491933 PMC5421560

[72] Qin K. Canonical and noncanonical Wnt signaling: Multilayered mediators, signaling mechanisms and major signaling crosstalk. Genes Dis 11, 103–134, doi:10.1016/j.gendis.2023.01.030 (2024). 37588235 PMC10425814

[73] Yu M. The evolving roles of Wnt signaling in stem cell proliferation and differentiation, the development of human diseases, and therapeutic opportunities. Genes Dis 11, 101026, doi:10.1016/j.gendis.2023.04.042 (2024). 38292186 PMC10825312

[74] Liao J. Long noncoding RNA (lncRNA) H19: An essential developmental regulator with expanding roles in cancer, stem cell differentiation, and metabolic diseases. Genes Dis 10, 1351–1366, doi:10.1016/j.gendis.2023.02.008 (2023). 37397543 PMC10311118

[75] Huang X. SATB2: A versatile transcriptional regulator of craniofacial and skeleton development, neurogenesis and tumorigenesis, and its applications in regenerative medicine. Genes Dis 9, 95–107, doi:10.1016/j.gendis.2020.10.003 (2022). 35005110 PMC8720659

[76] Luu H. H. Distinct roles of bone morphogenetic proteins in osteogenic differentiation of mesenchymal stem cells. J Orthop Res 25, 665–677, doi:10.1002/jor.20359 (2007). 17290432

[77] Pakvasa M. Notch signaling: Its essential roles in bone and craniofacial development. Genes Dis 8, 8–24, doi:10.1016/j.gendis.2020.04.006 (2021). 33569510 PMC7859553

